# Reducing financial barriers through the implementation of voucher incentives to promote children’s participation in community sport in Australia

**DOI:** 10.1186/s12889-019-8049-6

**Published:** 2020-01-07

**Authors:** L. J. Reece, C. McInerney, K. Blazek, B. C. Foley, L. Schmutz, B. Bellew, A. E. Bauman

**Affiliations:** 1 SPRINTER (The Prevention Research Collaboration’s Sport and Active Recreation Intervention & Epidemiology Research Group), Prevention Research Collaboration, School of Public Health, University of Sydney, D17 Charles Perkins Centre, Level 6, the Hub, Camperdown, NSW 2006 Australia; 2 Charles Perkins Centre, The University of Sydney, Camperdown, NSW Australia; 3 Biostatistics Training program NSW Ministry of Health Government, Sydney, Australia; 4 Australian Sports Commission, Canberra, Australia

**Keywords:** Participation, Children and young people, Sport, Physical activity, Voucher program

## Abstract

**Background:**

Participation in organised sport and physical activity contributes to health-enhancing levels of leisure time physical activity. In Australia, 58% of children aged 0–14 years participated at least once a week in October 2015 – December 2017. To overcome the frequently cited cost barrier, sports voucher incentives have been widely implemented across Australia.

**Method:**

The financial value of jurisdictional vouchers and the National median financial value were used to calculate the proportion of total annual expenditure on children’s participation in sport supported by sports vouchers. Participation rates using AusPlay data were estimated by age, sex and socio-economic index (SEIFA) at state and national level for children aged 0–14 years.

**Results:**

Five States and Territories implemented sports vouchers from 2011 to 2018, with a median value of AU$150. Nationally, median annual expenditure for children’s sport participation was AU$447 (IQR $194.2–936), with 27% reported expenditure supported by a sports voucher. The proportion of financial support from sports vouchers increased considerably with social disadvantage, rising to over 60% of total expenditure in the most disadvantaged populations.

**Conclusions:**

Socio-economic status was associated with sports-related expenditure and sports participation amongst children. Sport vouchers should target children in the most disadvantaged areas to promote participation in organised sport and physical activity.

## Background

Regular participation in physical activity has positive physical, emotional, social and mental health benefits [1] in children and adolescents [2]. To accrue benefits, global children’s physical activity guidelines recommend participation in at least 60 min of moderate intensity physical activity every day [3]. Whilst measurement of global child participation in physical activity remains complex [4] the global prevalence of adolescent physical inactivity is estimated at 80.3% (95%CI 80.1–80.5) with girls less active than boys [4]. Data from an Australian school based population survey of school with children aged 5 to 16 years data reinforces this, with only 1 in 5 children and adolescents (19%) meeting the daily physical activity recommendations [5]. Girls (15%) were less likely to meet the recommendation than boys (24%) along with children from urban areas (18%) and those from Middle Eastern (13%) and Asian cultural backgrounds (9%). Participation is frequently lower for children living in less affluent regions and who have inactive parents [6]. Less is known about the type of physical activities children participate in during leisure time [7] considering that active participation can occur in different physical activity domains, including sport.

Sport which is typically organised and structured, and played individually or within a team [1, 8] has been reported to produce various psychological and social health benefits that exceeds other forms of leisure time activity for children and adolescents [1, 8]. Sports club participation in Australia has been found to contribute significantly to leisure time activity [9] with clubs acting as the main avenue for both girls and boys under 15 years old to be active outside of the school environment. Despite the potential benefits, only 20% of children participate in organised sport outside of school hours at least three times per week [10]. Gender differences in participation are clear with boys most likely to continue to participate in sport throughout childhood whilst girls often see the competitive element of sports as a barrier to participation [11].

The main barriers to participating in physical activity and structured sport amongst children and young people include cost, accessibility, lack of parental support and a lack of local facilities [12, 13]. Associated with lower participation rates, families from the most disadvantaged areas are more substantially affected by these barriers. For example, families with low socio-economic status experience prohibitive costs associated with sporting registration, opportunities and equipment [14, 15].

Interventions to overcome cost barriers have been proposed with financial incentives and voucher programs receiving attention in adults. A systematic review of effectiveness of financial incentives used for promoting physical activity in the healthcare setting amongst adults found limited evidence and was inconclusive regarding their effectiveness on physical activity in this setting [16]. Lack of effectiveness was also the theme in research into the uptake and effectiveness of the Children’s Fitness Tax Credit in Canada (CFTC) [17]. Parents in the lowest income quartile were significantly less aware of and less likely to claim the CFTC than other income groups [17].Among parents who had claimed the CFTC, few (15.6%) believed it had increased their child’s participation in physical activity programs. It was concluded that whilst more than half of Canadian parents with children had claimed the CFTC, the tax credit scheme appeared to benefit wealthier families [17]. By contrast, a secondary school based voucher program in Wales, United Kingdom (UK), examined the effect of a multi-component voucher based intervention on the cardiovascular fitness and physical activity levels of teenagers aged 13–14 years in seven schools in Swansea (4 intervention and 3 control schools) called ACTIVE. This scheme positively influenced attitudes to physical activity and enabled children from disadvantaged backgrounds to access broader opportunities to participate in sport and physical activity [15].

With the inconclusive evidence on best practice strategies and the potential to achieve a 25% relative increase in population physical activity [18] the use of sports vouchers has been widely implemented across Australia. The aim of this paper is to examine Australian’s annual expenditure on children’s sport and assess the financial support achieved through sport vouchers across different states and territories, as a means of overcoming financial barriers and increasing Australian children’s participation in organised sport.

## Method

Data from AusPlay, the Australian sport sector’s national population participation assessment, were provided by the Australian Sports Commission [10]. The Computer Assisted Telephone Interviews (CATI) use overlapping dual frames of landline or mobile phones with 13 strata based on states, territories and greater capital city areas for continuous tracking all year long. Data reported here spanned the time period from October 2015 to December 2017 and included 7976 children with information on sport participation. Information on children was obtained from the child’s parent or guardian. Weights (Hughes et al., 2017) were calculated per quarter and adjusted for the number of quarters used in the analysis. A proxy measure for socio-economic status (SES) was calculated using the Australian Bureau of Statistics’ (ABS) Index of Relative Socio-economic Disadvantage (IRSD, 2016). The national percentiles of SES were used and were grouped into quartiles, with the first quartile corresponding to the most disadvantaged. Participation rates were estimated for Australia and by states/territories according to sex, age group or SEIFA quartile. Using the Sport sector definition in AusPlay, a participant was defined as someone who has participated in at least 1 session of organised sport in the preceding 12 months with sport related costs defined as money paid to the organisation or venue [18]. All analyses incorporated the complex survey design and were mainly done using SAS software (version 9.4, Copyright© 2016 by SAS Institute Inc., Cary, NC, USA) using SAS/STAT survey analysis procedures. For design based Mood’s test of medians, R 3.4.0 [19] and the survey package [20] were used.

A review of voucher schemes across Australian states and territories (Bellew and Young, 2017) provided detail on existence, delivery dates and voucher value. In brief here, The Sport Voucher scheme in Northern Territory, led by Northern Territory government and administered by the Department of Tourism Sport and Culture [21], provides children in urban areas to claim a $100 voucher twice a year and activities for remote children are coordinated by Regional Councils. At the time of publication, Victoria does not have a sport voucher scheme and Australian Capital Territory offered grants and other initiatives for being active [22]. KidSport in Western Australia enables children aged 5–18 years to participation in community sport and recreation [23]; FairPlay vouchers in Queensland focuses on children aged 5 to 17 years old [24] and Ticket to play in Tasmania is a governed funded program giving $100 to children aged 5–17 [25]. All schemes provide assistance with membership and registration fees.

The total annual cost of participation for children in each State/Territory were estimated. The value of vouchers for individual States and Territories and the national median were used to calculate the proportion of total annual expenditure supported by sports vouchers [21].

## Results

### Implementation of voucher schemes

Between 2011 and 2018, five out of the eight Australian State and Territory Governments have implemented a sports voucher scheme to children and adolescents aged between 0 and 18 years old. The median voucher value was AU$150 p.a. (range AU$50–200 p.a.).

### State and national participation rates

**Fig. 1:**
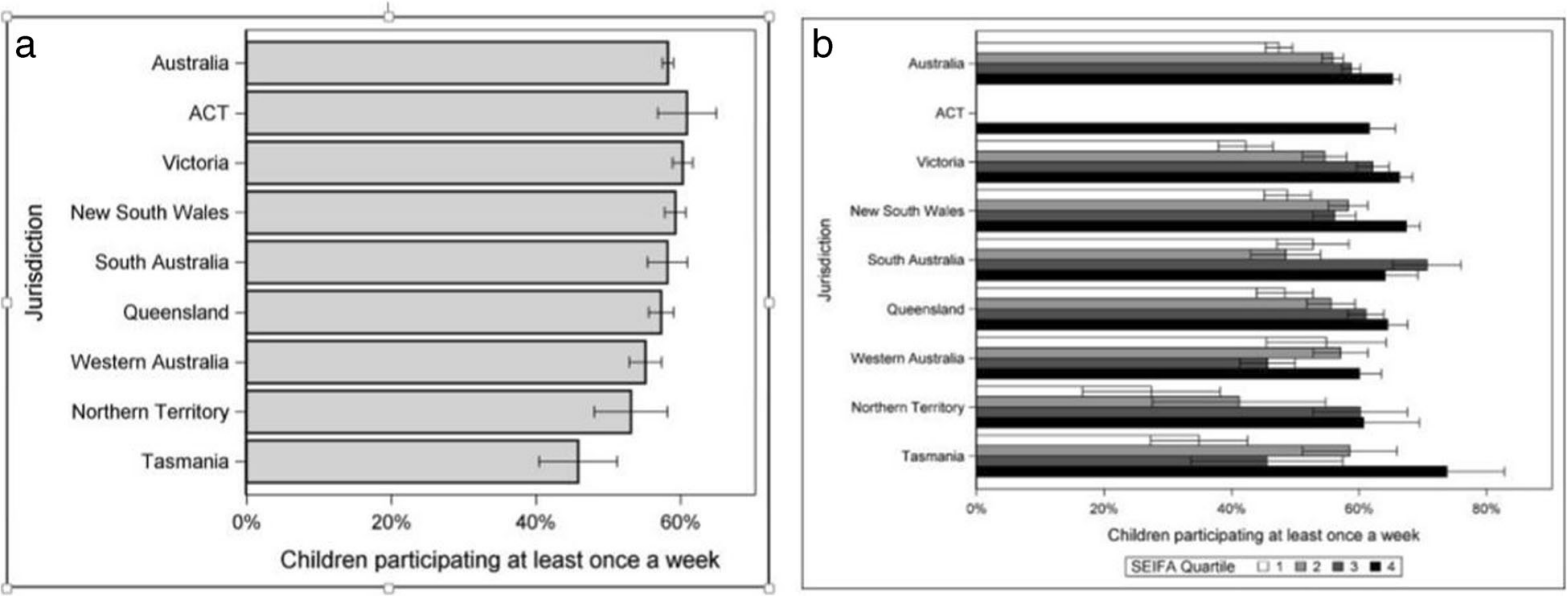
**a** Participation rates (at least once a week) by state. Bars represent standard error. **b** Participation rates (at least once a week) by SEIFA for all of Australia. Bars represent standard error

71% of Australian children took part in one or more organised sport or physical activity sessions outside of school hours, in the preceding 12 months. State variations exist with Tasmania at 61.2% ranging to Australian Capital Territory at 73.5%. Nationally, girls (70.6%) participation is slightly lower than boys (72.0%) yet this is not consistent across all states and territories, with girls exceeding the participation rates of boys in Tasmania and Victoria by 22 and 2.2% respectively. When assessing weekly participation in sport, national rates drop to 58.3% with boys and girls participation equal (see Fig. 1a). There is a continuous linear increase in participation both annually and weekly in areas of increased socio-economic advantaged. The difference nationally between the most and least disadvantaged areas in annual and weekly participation is 17% where those living in the most disadvantaged regions participated substantially less (see Fig. 1b).

### Individual expenditure on one child’s sport participation

Nationally, 94.8% of parents/carers with active children reported paying for their child’s participation in organised sport and physical activity outside of school hours in the last 12 months. Median annual expenditure of all activities was $447 (IQR $194.2–936), with girls participation costing more than boys ($103 difference in medians, *p* < 0.001) (Table 1). Median expenditure increases with age, from a median spend of $397 at 0–4 years and peaking at $479 amongst 9–11 year olds, although the test for medians across age groups was not significant (Table 1).

**Table 1:** Median yearly spend per child by sex, age group and SES. (IQR, interquartile range)

Category (group-wise *p*-value^a^)	Median	IQR
Sex (< 0.001)		
Male	396	177, 834
Female	499	197, 1037
Age (0.649)		
0–4	397	124, 777
5–8	445	177, 992
9–11	479	198, 1001
12–14	449	211, 900
SEIFA quartile (< 0.001)		
1 (most disadvantaged)	249	119, 651
2	369	158, 779
3	446	160, 935
4	560	248, 1151

^a^Design-based Mood’s test for the median

Socio-economic status influences total annual expenditure on sport with people living in the most disadvantaged communities spending less than half the amount spent by the least disadvantaged communities ($311 difference in medians, p < 0.001).

### Proportion of median annual expenditure supported by state voucher

**Fig. 2:**
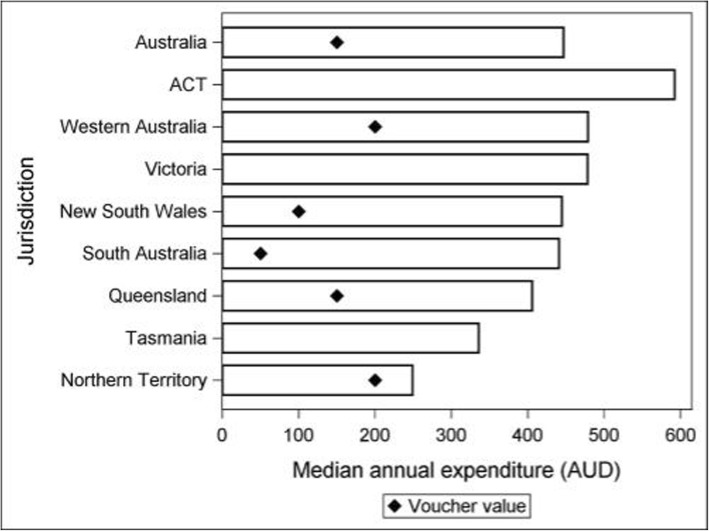
Median spend per child per year by state with value of state vouchers (where available) indicated with the diamond. The voucher value for Australia is the median value of the state vouchers (AUD150)

An assessment of the proportion of the median annual expenditure in each State met by their local sport voucher subsidy was conducted (Fig. 2). Northern Territory supported 80% of all costs, Western Australia 42%, Queensland 37%, NSW 23%, and South Australia 11% respectively. An inverse relationship is observed between expenditure and socio-economic status, with annual expenditure rising with decreasing disadvantage across all states and territories. The implementation of a voucher consistently supports a larger proportion of overall costs for children reporting lower expenditure

.

### Proportion of children’s sport expenditure supported by median Australian sport voucher value

To overcome state-wide variations, applying a median Australian voucher value, $150, identified that overall 34% of child sport related costs are supported, with marginal differences associated with gender and age. Applying the median national voucher value reinforces the bigger contribution to boys’ sports related costs (38%) and children aged 0–4 years (38%). A dose-response relationship exists between expenditure and SES, with more than half, (60%) of annual sport-related spend in the most disadvantaged communities supported by the implementation of a $150 voucher, compared to 27% least disadvantaged areas.

## Discussion

In the absence of national endorsement for the implementation of vouchers in Australia, most States and Territories have implemented state wide financial incentive schemes since 2011. Although each jurisdiction has taken a different approach either by voucher value or target audience, the principle consistently focused on the promotion of sport participation. This paper explored the effects of sport voucher programs at state and national level in Australia to reduce financial barriers to children’s sport participation. This analysis enhances existing evidence on family expenditure for sport [12]

Existing evidence infers the positive effect of financial incentives to increase physical activity amongst adults [16, 26]. Whilst frequently adopted in Australia, global comparisons are challenging, as their use is sparse with only selected countries including the United Kingdom, Luxembourg and Czech Republic, utilising this approach [15, 27, 28].Whilst the assessment of voucher program efficacy was not the purpose of this paper this was the first robust analysis to assess the proportion of individual sport-related expenditure supported by a voucher and its potential association with child participation rates, using the national population surveillance tool AusPlay, collectively analysed by age, sex and socio-economic status.

Nearly all parents of active children reported making annual payments for their child’s participation in organised sport and physical activity outside of school. National median expenditure for children aged 0–14 years was $447 (IQR 194.2–936), peaking in the 9–11 age category ($479; IQR 198.3–1001.2). Girls expenditure was consistently higher than boys ($499; IQR 197.4–1036.7) most likely, in light of girls preferred activities, such as dance which are frequently performed in specialised venues [10].

A previous narrative review [21] of voucher schemes explored their role in influencing participation and identified that within a multi-component strategy there was potential for a 25% relative increase in participation [26]. This, coupled with the expenditure data analysed here begins to describe the potential that a voucher scheme could contribute towards children’s physical activity levels.

The social inequalities in sport and physical activity amongst children and adolescents [8, 29] are reinforced through these analyses. Children in areas of low socio-economic status participated less frequently in organised sports and physical activity outside of school hours than their advantaged counterparts. A higher sporting club membership exists amongst higher income families [2, 8, 30] with children from advantaged communities more likely to receive greater logistical and financial support from the families to participate in consistent structured activity [31]. Disparities in the presence, geographic accessibility and affordability of sporting and recreational facilities have also been shown to influence child participation [8, 9]. Differences in the inclination towards sport can also be explained by economic factors as the traditional club based membership structures require financial outlay beyond sports club membership, including sports equipment and potential transportation costs [12, 29]. The dose response relationship between expenditure and socio-economic status was an important finding in this analysis, with a $311 difference in median expenditure between the most disadvantaged and least disadvantaged. The potential benefits of sport vouchers shows a social gradient, with 60% of current reported costs supported in the most disadvantaged areas. This is one step towards alleviating the financial barrier for disadvantaged communities to engage in sport. This finding is also reinforced at a State level, when applying individual state voucher values. For example NSW, the latest state to implement a voucher program, 36% sport related costs are supported by the $100 voucher in the most disadvantaged areas compared to only 19% in the least disadvantaged communities. As a result, it is important that whilst the implementation of universal voucher programs at state and national level offer potential to influence population participation, they must consider the specific targeting of priority groups including individuals from disadvantaged communities, children from CALD backgrounds, those in the overweight or obese body mass index categories, and children who are low active or completely inactive, to prevent a widening of the social gradient.

### Limitations

As participation is defined as at least one session of organised sport and physical activity outside of school hours in the last 12 months, it only represents a small fraction of health-related physical activity. Estimates of regular participation during leisure time are needed to learn more about the contribution of sport participation in overall leisure time physical activity. Further, parental report is used for most children’s organised sport and physical activity surveillance [9, 12, 32] and may have decreased reliability among adolescents.

## Conclusion

There is a clear opportunity for state-wide participation strategies, including the implementation of voucher programs, to promote participation in sport through their impact on financial costs of sport participation. Thorough evaluations of such programs are needed in order to understand the mechanisms which could result in larger-scale physical activity behavior change. Whilst understanding of the determinants remain unclear for sport, this paper reinforces the social gradient in child sport participation highlighting that the dose response relationship is also apparent in sport-related expenditure, with increasing expenditure with increasing advantage. It is therefore critical that the implementation of voucher programs focus on equitable and accessible participation by the specific targeting of of priority groups, including inactive and priority population, in order to reduce the gap between the least and most socioeconomically disadvantaged areas.

## Data Availability

The data that support the findings of this study are available from Australian Sports Commission but restrictions apply to the availability of these data, which were used under license for the current study, and so are not publicly available. Data are however available from the authors upon reasonable request and with permission of Australian Sports Commission.

## Abbreviations

ABS: Australian Bureau of Statistics
CATI: Computer Assisted Telephone Interviews
CFTC: Children’s Fitness Tax Credit in Canada
IRSD: Relative Socio-economic Disadvantage
SEIFA: Socio-economic Indexes for Areas
SES: Socio-economic status
UK: United Kingdom
